# Role of *POLE* and *POLD1* in familial cancer

**DOI:** 10.1038/s41436-020-0922-2

**Published:** 2020-08-14

**Authors:** Pilar Mur, Sandra García-Mulero, Jesús del Valle, Lorena Magraner-Pardo, August Vidal, Marta Pineda, Giacomo Cinnirella, Edgar Martín-Ramos, Tirso Pons, Adriana López-Doriga, Sami Belhadj, Lidia Feliubadaló, Pau M. Munoz-Torres, Matilde Navarro, Elia Grau, Esther Darder, Gemma Llort, Judit Sanz, Teresa Ramón y Cajal, Judith Balmana, Joan Brunet, Victor Moreno, Josep M. Piulats, Xavier Matías-Guiu, Rebeca Sanz-Pamplona, Rosa Aligué, Gabriel Capellá, Conxi Lázaro, Laura Valle

**Affiliations:** 1 Hereditary Cancer Program, Catalan Institute of Oncology, IDIBELL, Hospitalet de Llobregat, Barcelona, Spain; 2 Program in Molecular Mechanisms and Experimental Therapy in Oncology (Oncobell), IDIBELL, Hospitalet de Llobregat, Barcelona, Spain; 3 Centro de Investigación Biomédica en Red de Cáncer (CIBERONC), Madrid, Spain; 4 Oncology Data Analytics Program (ODAP), Catalan Institute of Oncology, IDIBELL, Hospitalet de Llobregat, Barcelona, Spain; 5 Prostate Cancer Clinical Research Unit. Spanish National Cancer Research Center (CNIO), Madrid, Spain; 6 Department of Pathology, Bellvitge University Hospital, IDIBELL, Hospitalet de Llobregat, Barcelona, Spain; 7 Department of Biomedical Sciences, School of Medicine, University of Barcelona, IDIBAPS, Barcelona, Spain; 8 Department of Immunology and Oncology, National Center for Biotechnology (CNB-CSIC), Spanish National Research Council, Madrid, Spain; 9 Centro de Investigación Biomédica en Red de Epidemiologia y Salud Pública (CIBERESP), Madrid, Spain; 10 Hereditary Cancer Program, Catalan Institute of Oncology, IDIBGi, Girona, Spain; 11 Department of Medical Oncology, Parc Taulí, Hospital Universitari Parc Taulí, Sabadell, Barcelona, Spain; 12 Consorci Sanitari de Terrassa, Terrasa, Barcelona, Spain; 13 Genetic Counseling Unit, Medical Oncology Department, Althaia Xarxa Assistencial Universitària de Manresa, Manresa, Spain; 14 Department of Medical Oncology, Hospital de la Santa Creu i Sant Pau, Barcelona, Spain; 15 Department of Medical Oncology, Vall d’Hebron University Hospital, Vall d’Hebron Institute of Oncology (VHIO), Barcelona, Spain; 16 Department of Clinical Sciences, Faculty of Medicine, University of Barcelona, Barcelona, Spain; 17 Department of Medical Oncology, Bellvitge University Hospital, IDIBELL, Hospitalet de Llobregat, Barcelona, Spain

**Keywords:** polymerase proofreading–associated polyposis, PPAP, exonuclease domain, ultramutated phenotype, hereditary colorectal cancer

## Abstract

**Purpose:**

Germline pathogenic variants in the exonuclease domain (ED) of polymerases *POLE* and *POLD1* predispose to adenomatous polyps, colorectal cancer (CRC), endometrial tumors, and other malignancies, and exhibit increased mutation rate and highly specific associated mutational signatures. The tumor spectrum and prevalence of *POLE* and *POLD1* variants in hereditary cancer are evaluated in this study.

**Methods:**

*POLE* and *POLD1* were sequenced in 2813 unrelated probands referred for genetic counseling (2309 hereditary cancer patients subjected to a multigene panel, and 504 patients selected based on phenotypic characteristics). Cosegregation and case–control studies, yeast-based functional assays, and tumor mutational analyses were performed for variant interpretation.

**Results:**

Twelve ED missense variants, 6 loss-of-function, and 23 outside-ED predicted-deleterious missense variants, all with population allele frequencies <1%, were identified. One ED variant (*POLE* p.Met294Arg) was classified as likely pathogenic, four as likely benign, and seven as variants of unknown significance. The most commonly associated tumor types were colorectal, endometrial and ovarian cancers. Loss-of-function and outside-ED variants are likely not pathogenic for this syndrome.

**Conclusions:**

Polymerase proofreading–associated syndrome constitutes 0.1–0.4% of familial cancer cases, reaching 0.3–0.7% when only CRC and polyposis are considered. ED variant interpretation is challenging and should include multiple pieces of evidence.

## INTRODUCTION

Germline missense pathogenic variants in the exonuclease domain (ED) of polymerases epsilon (*POLE*; a.a. 268–471) and delta (*POLD1*; a.a. 304–533), which affect the proofreading capabilities of these polymerases, predispose to multiple colorectal adenomas and carcinomas, causing the so-called polymerase proofreading–associated polyposis (PPAP) (MIM 615083; 612591).^1–7^ Evidence of extracolonic tumors has been reported, including endometrial, brain, breast, ovarian, stomach, pancreas, and skin tumors, among others.^2,6–10^

Somatic pathogenic variants in the ED of *POLE* have been identified in 2–8% of colorectal cancer (CRC),^11–14^ 7–15% of endometrial tumors,^15–18^ and more rarely in other tumor types.^19–21^ Somatic pathogenic variants affecting *POLD1* ED are extremely rare. Tumors with somatic *POLE* ED pathogenic variants and tumors developed in the context of PPAP exhibit a dramatically increased mutation rate known as ultramutated tumor phenotype,^17,21–23^ characterized by a specific mutation signature typified by C:G→A:T transversions (TCT context) and C:G→T:A transitions (TCG context).^12,24^ This corresponds to signature 10 in the COSMIC Signatures of Mutational Processes in Human Cancer, or to signatures 14 and 20 if the *POLE* or *POLD1* pathogenic variant coexists with mismatch repair (MMR) deficiency, respectively.^12,24,25^ Cancer patients with somatic *POLE* ED pathogenic variants show excellent prognosis and good response to immune checkpoint inhibition, probably due to the immune response elicited by these tumors as a result of the large number of neopeptides generated as consequence of hypermutation.^26^

To assess the prevalence of *POLE* and *POLD1* pathogenic variants in hereditary cancer and refine the tumor spectrum of the associated clinical syndrome, we studied a prospective cohort of 2309 unrelated hereditary cancer patients subjected to a multigene hereditary cancer panel, and a retrospective cohort of 504 unrelated cancer patients—hereditary CRC and polyposis patients excluded—selected based on previous reports of extracolonic manifestations in PPAP, which include breast and ovarian cancer, endometrial, brain, or skin cancer, among other tumors, alone or in combination with other tumor types or colonic polyps. Cosegregation analyses, yeast-based assays, and tumor mutational analyses were performed to facilitate variant interpretation.

## MATERIALS AND METHODS

### Ethics statement

Patients signed consent forms and were assessed at genetic counseling units at Catalan Institute of Oncology, Vall d’Hebron Institute of Oncology, and Santa Creu i Sant Pau, Parc Taulí and Manresa Hospitals (Catalonia, Spain). The study received the approval of IDIBELL Ethics Committee.

### Patients

#### Retrospective cohort

Five hundred and four unrelated cancer patients were analyzed, including 192 high risk breast and/or ovarian families, 178 patients with personal or familial history of different tumor types previously associated with PPAP (combinations include CRC and associated tumors, breast/ovarian cancer, skin cancer or brain), 30 patients with aggregation of other multiple tumors, either in the patient or the family, and finally, 104 patients fulfilling the criteria for *TP53* genetic testing. None of the patients carried germline pathogenic variants in the known high-penetrance cancer genes associated with the patient and/or family’s phenotype (Supplementary Table S1).

#### Prospective cohort

Two thousand three hundred and nine unrelated familial/early-onset cancer patients, prospectively recruited from 2015 to 2018 in a hereditary cancer clinic–based context, were subjected to a multigene hereditary cancer panel.^27,28^ The cohort comprised patients with personal and/or familial history of breast cancer (*n* = 884), ovarian cancer (*n* = 317), and breast and ovarian cancer (*n* = 267); 247 familial (attenuated and classic) adenomatous polyposis cases; 354 Amsterdam- or Bethesda-positive hereditary nonpolyposis CRC cases; 15 Li–Fraumeni–suspected cases; and 225 patients with suspicion of other minor cancer syndromes.

**Fig. 1:**
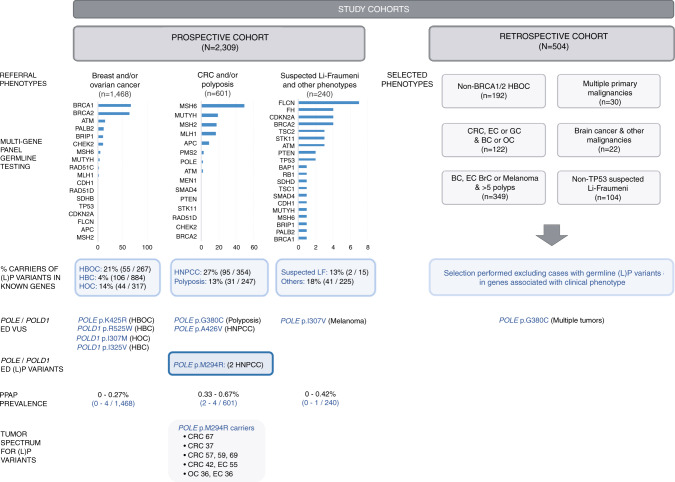
Schematic representation of the characteristics of the cohorts analyzed in this study, prevalence of (likely) pathogenic variants in cancer-predisposing genes, and results of the current study for exonuclease domain (ED) missense variants in *POLE* and *POLD1*. *BC* breast cancer, *BrC* brain tumor, *CRC* colorectal cancer, *EC* endometrial cancer, *GC* gastric cancer, *HBC* hereditary breast cancer, *HBOC* hereditary breast and ovarian cancer, *HOC* hereditary ovarian cancer, *HNPCC* hereditary nonpolyposis colorectal cancer, *LF* Li–Fraumeni syndrome, *LP* likely pathogenic, *OC* ovarian cancer, *PPAP* polymerase proofreading–associated polyposis, *VUS* variant of unknown significance.

Details of the cohorts are included in Fig. 1 and Supplementary Table S1.

### Mutational screening

In the retrospective cohort, direct automated (Sanger) sequencing was used to sequence exons 8–13 (+/−20 b) of *POLD1* and exons 9–14 (+/−20 b) of *POLE*, which contain the sequences coding for the ED of each polymerase. Primer sequences were previously described.^7^ The hereditary cancer multigene panel applied to the prospective cohort includes the complete coding sequence of *POLE* and *POLD1*.^27,28^ Nonsynonymous variants located within coding exons or variants affecting canonical splice sites with a population minor allele frequency (MAF) <1% were considered.

### In silico predictions

The pathogenicity of the identified missense variants was analyzed by using the metapredictor REVEL, which combines pathogenicity predictions and conservation information obtained from 18 individual scores, and provides optimal specificity and sensitivity results.^29,30^ REVEL score ≥0.35 was used for pathogenicity in the current study, a lower cutoff than the one recommended for clinical purposes (≥0.5) and defined based on the scores of known ED pathogenic variants (Supplementary Table S2).

### Functional assessments for variant interpretation and classification

The exonuclease repair ability of *POLE* in the presence of ED missense variants was tested in a *Schizosaccharomyces pombe* system. Exome sequencing was performed on DNA extracted from the tumors of carriers of *POLE/D1* variants to assess the presence of hyper or ultramutation (10 or 100 Mut/Mb respectively) and/or the mutational signature(s) associated with the presence of an ED pathogenic variant.^24,31^ *POLE* and *POLD1* variant frequencies in CRC and controls were obtained from a Spanish population-based case–control study (MCC-Spain, www.mccspain.org). ED variants were classified by applying the American College of Medical Genetics and Genomics/Association for Molecular Pathology (ACMG/AMP) guidelines^32^ (Supplementary Table S3). Additional details are in Supplementary Methods.

## RESULTS

Of the 2309 unrelated probands included in the prospective cohort, 374 (16.2%) carried (likely) pathogenic germline variants in known cancer-predisposing genes (Fig. 1; Supplementary Table S4).

A total of 12 novel or rare (MAF_gnomAD_<1%) missense variants located within the EDs of *POLE* (*n* = 7) or *POLD1* (*n* = 5) were identified in 15 families (Table 1). Five loss-of-function (LoF) variants were found in five families, and 23 predicted-deleterious *POLE* (*n* = 19) and *POLD1* (*n* = 4) variants outside the ED were identified in 29 probands (Supplementary Fig. S1).

**Table 1:** *POLE* and *POLD1* exonuclease domain germline variants identified in the study (population MAF <1%).

Genetic variant (motif/DNA binding cleft) (Suppl. Fig. S1 and Fig. 2)^a^	dbSNP rs # population MAF_NFE_ (%)^b^	Evolutionary conservation (PhyloP/PhastCons)^c^ In silico prediction (REVEL)^d^	Proband’s phenotype and cosegregation data (Suppl. Figs. S2 and S3)	Mutator phenotype in yeast (Fig. 2)^e^	Tumor sequencing analysis (mutational burden and signature contribution) (Fig. 3 and Suppl. Tables S6 and S7)	ACMG/AMP variant classification^f^^42^ (InterVar/^g^InterVar-adjusted)
***POLE***						
c.861T>A; p.Asp287Glu (Flanking Exo I/Yes)	rs139075637 (0.1710)^h^	Moderately conserved (1.189/1) Benign (0.286)	**Fam A:** BC 40 *Noncarrier:* Mother (OC 53) **Fam B:** BC 59; CRC 59 *Cancer-affected carriers vs. total cancer-affected individuals:*^i^ 7/13 (2/6 in one same family)	Yes (+++)	**Fam A–II.3 (BC):** 185.7 Mut/Mb; 16.3% MMR-d sig	**Likely benign** (PM1, PM2, BS2 and BP4)/**Likely benign** (PM1, BP4, PS3_moderate, BS1, BS4_supporting)
c.881T>G; p.Met294Arg (Flanking Exo I/Yes)	n.a.	Conserved (9.279/1) Damaging (0.816)	**Fam C:** OC 36; EC 36 *Carriers:* mother (CRC 42; EC 55), uncle (CRC 67) **Fam D:** CRC 37 *Carriers:* uncle (CRC 57, 59, 69) *Total meiosis*: 3 across 1 family	Yes (++)	**Fam C–II.2 (OC):** 249 Mut/Mb; 15.3% MMR-d + POLE sig (SBS14) **Fam C–II.2 (EC):** 300 Mut/Mb; 95.4% MMR-d sigs **Fam C–I.4 (CRC):** 11 Mut/Mb; 23.1% POLE sig (SBS10)	**Uncertain significance** (PM1, PM2)/**Pathogenic** (PM1, PM2, PP1, PP3, PS3_supporting, PP4_strong)
c.919A>G; p.Ile307Val (outside Exo/No)	n.a.	Conserved (7.979/1) Benign (0.337)	**Fam E:** Melanoma (×4) 81 *Carriers:* sister (BC 83), sister (melanoma 55)	Yes	**Fam E–II.1 (Melanoma):** 32 Mut/Mb; 21.6% MMR-d sigs	**Uncertain significance** (PM1, PM2)/(PM2)
c.1007A>G; p.Asn336Ser (outside Exo/No)	rs5744760 (0.0197)^j^	Conserved (7.979/1) Damaging (0.425)	**Fam F:** CRC 38 **Fam G:** Melanoma 48	No effect^40^	n.a.	**Benign** (PM1, BS1, BS2 and BP6)/**Likely benign** (BS1, BS3_supporting, PP3)
c.1138G>T; p.Gly380Cys (outside Exo/No)	rs199746481 (0.0051)	Conserved (7.867/0.878) Damaging (0.531)	**Fam H:** BC (×2) 61 **Fam I:** 10–20 Polyps 72	Yes (++)	**Fam H–II.4 (BC):** 0.7 Mut/Mb	**Uncertain significance** (PM1, PM2, PP3)/(PP3, PS3_supporting)
c.1274A>G; p.Lys425Arg (Exo IV/Yes)	rs757186755 (0.0025)	Conserved (7.979/1) Damaging (0.457)	**Fam J:** BC 46; OC 48, EC 48 Total reported carriers:^k^ 8	n.p.	n.a.	**Uncertain significance** (PM1 and PM2)/(PM1, PP3)
c.1277C>T; p.Ala426Val (Exo IV/Yes)	rs374920539 (0.0034)	Conserved (9.953/1) Damaging (0.392)	**Fam K:** Renal cancer 49; CRC 44; adenomas. *Carriers:* paternal uncle (CRC 73), father (CRC 60) *Total meiosis*: 3 across 1 family	Yes (++)	**Fam K–II.1 (CRC):** 41 Mut/Mb; 16.3% MMR-d sigs	**Uncertain significance** (PM1 and PM2)/(PM1, PP3, PS3_supporting, PP1)
***POLD1***						
c.921T>G; p.Ile307Met (outside Exo/No)	n.a.	Nonconserved (−2.516/0.00) Benign (0.268)	**Fam L:** OC 40	Not conserved in *S. pombe*	n.a.	**Uncertain significance** (PM1, PM2 and BP4)/(PM2 and BP4)
c.973A>G; p.Ile325Val (Exo I/Yes)	rs558345043 (0.0017)	Moderately conserved (2.440/0.984) Benign (0.041)	**Fam M:** BC 33	Not conserved in *S. pombe*	n.a.	**Uncertain significance** (PM1, PM2, and BS2)/(PM1, BP4)
c.1054C>T; p.Arg352Cys (outside Exo/No)	rs762330164 (0.0032)	Conserved (4.348/1) Benign (0.299)	**Fam N:** 5 adenomatous polyps	n.p.	**TCGA (CRC):** 35 Mut/Mb, 71% MMR-d sigs. **COSMIC (Lymph):** 24.7 Mut/Mb; non-*POLE/D1*, non-MMR-d sigs	**Uncertain significance** (PM1 and PM2)/**Likely benign** (BP4, BP5)
c.1562G>A; p.Arg521Gln (outside Exo/Yes)	rs143076166 (0.0238)^l^	Conserved (7.044/1) Benign (0.278)	**Fam O:** CRC (×2) 34	n.p.	**Fam-6 (CRC)**^7,43^**:** 39 Mut/Mb; 14.5% MMR-d sigs (14.51%)	**Uncertain significance** (PM1 and PM2)/**Likely benign** (PM1, BS2_supporting and BP4)
c.1573C>T; p.Arg525Trp (outside Exo/Yes)	rs201804732 (0.0010)	Moderately conserved (3.030/1) Benign (0.267)	**Fam P:** BC 35, 49, 53; CRC 69	Not conserved in *S. pombe*	**TCGA (GC):** 13.7 Mut/Mb; non-*POLE/D1*, non-MMR-d sigs (other DNA repair defects)	**Uncertain significance** (PM1,PM2 and BS2)/(PM1, PM2, BP4)

*ACMG/AMP* American College of Medical Genetics and Genomics/Association for Molecular Pathology, *CRC* colorectal cancer, *EC* endometrial cancer, *GC* gastric cancer, *gnomAD* Genome Aggregation Database, *Lymph* lymphoid neoplasm, *MAF* minor allele frequency, *MMR-d* mismatch repair deficiency, *n.a.* not available; *NFE* non-Finnish Europeans, *n.p.* not performed, *OC* ovarian cancer, *SBS* single base signature, *sig(s)* signature(s).

^a^RefSeq GRCh37: *POLE* (NM_006231.2; NP_006222.2) and *POLD1* (NM_001256849.1; NP_001243778.1).

^b^Population MAF obtained from gnomAD non-Finnish, noncancer Europeans (gnomAD v.2.1.1).

^c^PhyloP/PhastCons values were obtained from alignments of 100 vertebrate sequences. The higher the score, the more conserved the site is (PhyloP range score: −20 to +10; PhastCons: 0 to 1).

^d^In silico prediction according to REVEL ≥0.35.

^e^Yeast variant rate increase (+, *p* = 0.01; ++, *p* = 0.01–0.001; +++, *p* < 0.001 indicate the differences with the wildtype control, calculated using the Mann–Whitney nonparametric test).

^f^According to the standard guidelines for the clinical interpretation of genetic variants (ACMG/AMP recommendations) using the automatic InterVar software.

^g^ACMG/AMP–based manual curation (new evidence considered is underlined). Details are shown in Supplementary Methods and Supplementary Table S3: PM2, absent or <1/100,000 in general population (gnomAD noncancer data set); PP1, cosegregation with disease in multiple affected family members (at least three meiosis across one family); PP3, in silico prediction indicates that the variant could be damaging (REVEL ≥ 0.35); PP4, the patient’s phenotype is highly specific for a disease with a single genetic etiology (moderate: when hyper/ultramutation and *POLE/D1*-associated mutational signatures 14 or 20 were detected in the tumor; strong: when hyper/ultramutation and the *POLE/D1*-associated mutational signature 10 were detected in the tumor); PS3, functional studies are supportive of a damaging effect on the gene (supporting: when a mutator phenotype in *S. pombe* was observed with *p* = 0.01–0.001; moderate: when a mutator phenotype in *S. pombe* was observed with *p* < 0.001). BS1, allele frequency is greater than expected for disorder (considering MAF > 0.1% in any gnomAD v2.1.1 noncancer subpopulation); BS2_supporting, observed in ≥10 healthy adult individuals (above 60 years of age) for a dominant (heterozygous) disorder (not applied when BS1 was considered); BS3, functional studies showed no damaging effect on protein function (supporting: when a mutator phenotype in *S. pombe* was not observed); BS4_supporting, lack of segregation in more than three cancer-affected members (same or related phenotype) of a family; BP4, in silico prediction of pathogenicity estimated by REVEL suggests no impact (REVEL < 0.30). BP5, variant found in a case with an alternate molecular basis for disease (for tumors with somatic *POLE/D1* variants: when an MMR-proficient tumor was not hyper/ultramutated or when an DNA repair–proficient tumor was hypermutated but mutational signatures 10, 14, or 20 were not identified. For tumors from carrier families: same criteria as defined in the previous sentence for tumors with somatic variants but applied to two different tumors to avoid a false classification due to the presence of a phenocopy).

^h^MAF in European non-Finnish, noncancer population (gnomAD): 202/118,154 individuals (60 above 60 years of age).

^i^Characteristics of *POLE* c.861T>A (p.Asp287Glu) carrier families are shown in Supplementary Table S4.

^j^MAF in African, noncancer population (gnomAD): 623/23,530 individuals (2.6%); 10 homozygous carriers.

^k^*POLE* c.1274A>G (p.Lys425Arg) carriers from our study and previously reported.^3,10,35,36^

^l^MAF in European non-Finnish, noncancer population (gnomAD): 28/117,648 individuals (10 above 60 years of age).

Families carrying the corresponding *POLE/POLD1* variant, tumors studied for the analysis of mutational burden and signatures, and ACMG/AMP variant classification results, are highlighted in bold. Cosegregation data (carriers, non-carriers, meiosis) are shown in italics.

Mutational screening of the region coding the ED of *POLE* and *POLD1* in the retrospective cohort (*n* = 504 unrelated probands) identified a missense *POLE* ED variant, c.1138G>T (p.Gly380Cys), and a frameshift variant, *POLE* c.1185_1188del (p.Glu396Thrfs*15).

### Phenotypic characteristics of carriers of ED variants

Twelve rare or novel missense variants located within the EDs of *POLE* (*n* = 7) and *POLD1* (*n* = 5) were identified in 16 unrelated individuals of the 2813 cancer patients studied (Table 1; pedigrees in Supplementary Figs. S2 and S3).

*POLE* c.861T>A (p.Asp287Glu) (MAF_gnomAD_NFE_ = 0.17%) was identified in two unrelated breast cancer patients, one of whom was also diagnosed with an MMR-deficient CRC (Table 1). This variant had been previously reported in three unrelated families diagnosed with melanoma at ages 22–73 and other tumors, including breast cancer, squamous cell carcinoma, and non-Hodgkin lymphoma,^10,33^ and in a patient with a Lynch syndrome–associated *MLH1*-deficient colorectal tumor (Supplementary Table S5).^34^ Of the 13 studied cancer-affected individuals (in this and previous reports) only 7 carried *POLE* c.861T>A. No association with cancer was detected for this variant in a Spanish case–control study, either for CRC or breast cancer (source: MCC-Spain; Supplementary Table S6).

The novel, predicted pathogenic *POLE* c.881T>G (p.Met294Arg) variant, which affects a highly conserved amino acid, was identified in two families. One proband was a woman diagnosed with ovarian and endometrial tumors at age 36. Her mother, diagnosed with CRC and endometrial cancer at ages 42 and 55 respectively, and her CRC-affected uncle, were also carriers. *POLE* c.881T>G was also identified in a patient diagnosed with an MMR-proficient CRC at age 37, and in his maternal uncle, affected with three metachronous CRCs. The variant has been recently reported in a patient affected with multiple colonic adenomas, breast cancer, and endometrial cancer, diagnosed at 48, 50, and 55 years old respectively, and in her sister, with seven colonic adenomas at age 58.^35^

*POLE* c.919A>G (p.Ile307Val), affecting a conserved residue and not reported in public databases, was identified in an individual with four melanomas diagnosed at age 81 and in two of his sisters, diagnosed with melanoma at 55 and breast cancer at 83.

*POLE* c.1007A>G (p.Asn336Ser) (MAF_gnomAD_NFE_ = 0.02%) was identified in two families. One of the probands, of Moroccan origin, was diagnosed with rectal cancer at age 38, and the other one with melanoma at 48. This variant is relatively frequent in African populations (MAF = 2.6%), where a total of 10 homozygous carriers have been identified among 11,975 Africans (gnomAD v.2.1.1).

*POLE* c.1138G>T (p.Gly380Cys) (MAF_gnomAD_NFE_ = 0.005%), predicted deleterious, was identified in a woman diagnosed with two synchronic breast tumors, and in an unrelated individual with multiple (10–20) adenomatous polyps and no familial cancer history.

*POLE* c.1274A>G (p.Lys425Arg) (MAF_gnomAD_NFE_ = 0.0025%) was identified in a woman affected with breast, ovarian, and endometrial tumors before age 50. This predicted pathogenic variant affects a highly conserved residue located within the Exo IV motif active site. The variant had been previously reported in six families, including a melanoma patient,^10^ a patient with early-onset CRC,^3^ an individual with colonic polyps and family history of CRC,^36^ a 63-year-old CRC patient,^35^ a woman diagnosed with breast cancer at age 31,^35^ and two first-degree relatives, who also carried the *MSH2* c.942+3A>T pathogenic variant, diagnosed with three synchronic CRCs at age 30 and one CRC at age 33 respectively.^35^

*POLE* c.1277C>T (p.Ala426Val) (MAF_gnomAD_NFE_ = 0.0034%), affecting a highly conserved residue within the Exo IV motif active site, was identified in a male diagnosed with CRC and multiple adenomas at age 44 and with renal cancer at 49, and in his CRC-affected paternal uncle. The proband’s father, an obligate carrier, was diagnosed with CRC at age 60. All three relatives also carried a likely pathogenic variant in *CHEK2*, c.593–1G>T (rs786203229), a gene that confers a moderate risk of breast cancer, and possibly other tumor types, including CRC.^37,38^ In this family, the carriers of the two variants did not develop particularly aggressive phenotypes, two of them having developed CRC late in life (ages 60 and 73).

The five ED variants identified in *POLD1*, p.Ile307Met, p.Ile325Val, p.Arg352Cys, p.Arg521Gln, and p.Arg525Trp, were predicted to be neutral (Table 1; pedigrees in Supplementary Fig. S3). *POLD1* c.1562G>A (p.Arg521Gln) had been previously identified by our group in a 48-year-old CRC patient.^7^ *POLD1* c.1573C>T (p.Arg525Trp) co-occurred with *ATM* c.7220C>A (p.Ser2407*; rs1555122149) in a woman affected with multiple tumors. *ATM* pathogenic variants confer a moderate risk of breast cancer (two to fivefold), but are not associated with bilateral breast tumors.^37,38^ Available studies have been unable to quantify the postulated increased risk to CRC.^39^ The carrier of *POLD1* p.Arg525Arg and *ATM* p.Ser2407* developed a very aggressive phenotype, including three metachronous early-onset breast tumors (ages 35–53) and a CRC at age 69, supporting a cumulative effect of the two variants.

### Functional assessment of ED variants: exonuclease repair yeast-based assay and tumor analysis

**Fig. 2:**
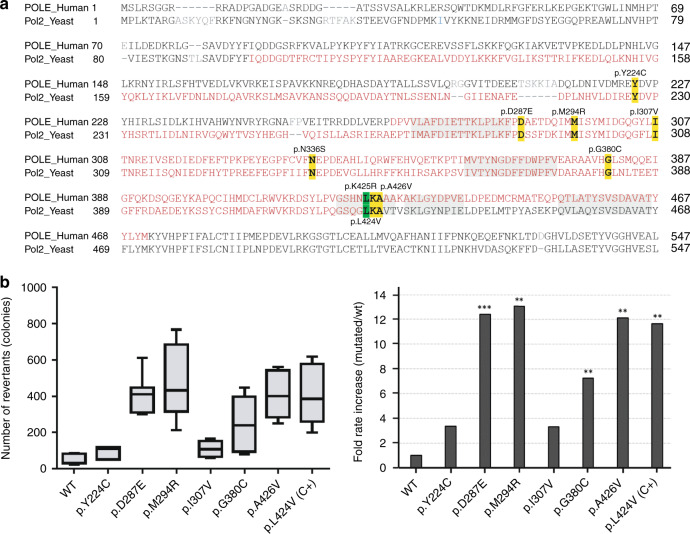
Functional analysis carried out in *Schizosaccharomyces pombe* for variants located within (or close to) the *POLE* exonuclease domain (ED). (**a**) Alignment of human *POLE* and their homolog in yeast (*Pol2*). The identified variants are highlighted in yellow (conserved residues) and the *POLE* p.Leu424Val, used as positive control, in green. ED is depicted in red (human: residues 268–471, yeast: residues 98–428) and its sequence motifs^44^ are shaded in gray. (**b**) Left panel: box plots showing mutation rates of ade6-485 *S. pombe* (number of colonies) for *pol2* wildtype (WT, negative control), ED mutation-positive control (*pol2*-Leu425Val; C+) and the corresponding variants. Right panel: fold rate increase relative to the median number of revertants in the WT. Data obtained from two independent experiments performed in triplicate. *p value = 0.01, **p value = 0.01–0.001, and ***p value < 0.001 indicate the differences with the WT clone, and were calculated using the Mann–Whitney nonparametric test.

**Fig. 3:**
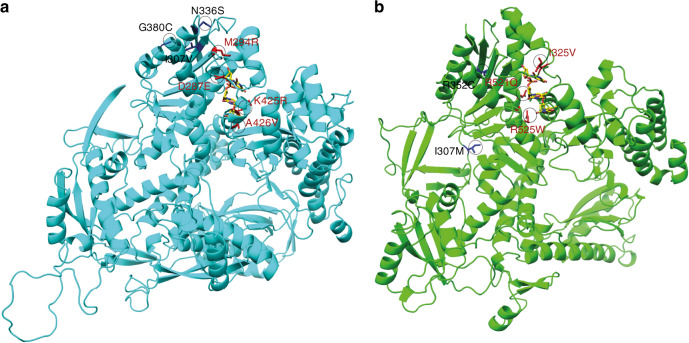
Structural representation of human POLE and POLD1 and location of the ED variants identified in the current study. (**a**) 3D structure of POLE. (**b**) 3D structure of POLD1. Single-stranded DNA from the aligned bacteriophage T4 polymerase complex (PDB ID: 1NOY) is shown in yellow. Variants in the DNA binding pocket are highlighted in red.

The exonuclease repair ability of *POLE* in the presence of missense ED variants was tested in a yeast system. The number of revertant colonies was significantly higher for p.Leu424Val (positive control), p.Asp287Glu, p.Met294Arg, p.Gly380Cys, and p.Ala426Val, compared with the wildtype (fold change increase of 7–13) (Fig. 2). No significant differences were observed for *POLE* p.Ile307Val, and a previous study had shown no effect for p.Asn336Ser.^40^ Notably, *POLE* p.Asp287Glu, p.Met294Arg, p.Leu424Val, and p.Ala426Val, the ones with the highest number of revertant colonies, were located within the DNA binding pocket structure (Fig. 3). All *POLD1* ED variants identified in the study were predicted benign, and only two, p.Arg352Cys and p.Arg521Gln, affected conserved amino acids in yeast. Since all gathered evidence for those two was enough to classify them as likely benign (Table 1), we did not perform the yeast-based functional assay.

**Fig. 4:**
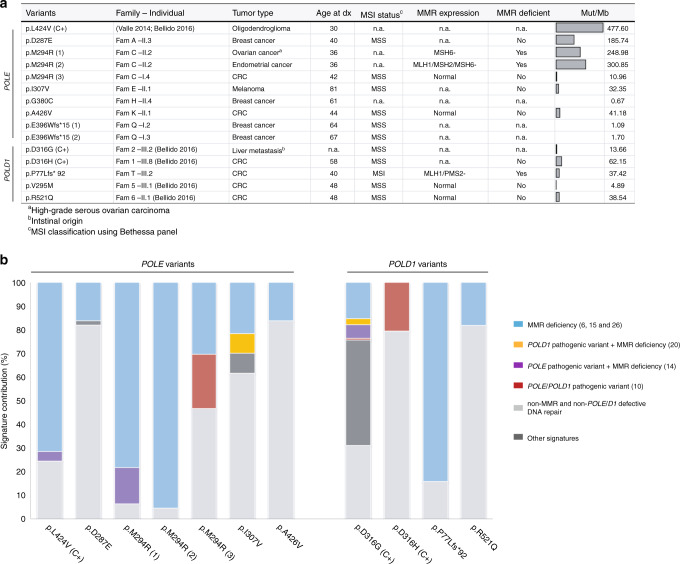
Somatic analysis performed in tumors from *POLE/POLD1* variant carriers. (**a**) Tumor features including mismatch repair (MMR) deficiency status and mutational burden (hypermutation was considered when the tumor had more than 10 exonic Mut/Mb). ^a^High-grade serous ovarian cancer. ^b^Intestinal origin. ^c^Microsatellite instability (MSI) classification using Bethesda panel. C+, positive controls, i.e., tumors from carriers of variants affecting the *POLE/POLD1* ED previously classified as pathogenic. (**b**) Mutational signature contribution (DeconstructSigs) for hyper/ultramutated tumors (>10 Mut/Mb). *CRC* colorectal cancer.

Exome sequencing was performed in tumors developed by *POLE* p.Asp287Glu, p.Met294Arg, p.Ile307Val, p.Gly380Cys, p.Ala426Val, and *POLD1* p.Arg521Gln carriers, including, as positive controls, tumors developed by *POLE* p.Leu424Val, *POLD1* p.Asp316Gly, and *POLD1* p.Asp316His carriers^2,7^ (Fig. 4; Supplementary Table S7). All positive controls showed hyper- or ultramutation (range: 14–478 Mut/Mb). *POLE/D1* ED-associated signatures were detectable in the *POLD1* p.Asp316His carrier’s CRC tumor (signature 10 contribution: 20.66%), but barely identifiable in the *POLD1* p.Asp316Gly carrier’s liver metastasis (signature 10: 0.75%; signature 20 [*POLD1* + MMR deficiency]: 2.65%) or in the *POLE* p.Leu424Val carrier’s oligodendroglioma (signature 14 [*POLE* + MMR deficiency]: 4%), where MMR deficiency hoarded most of the mutational signatures’ contribution.

Three primary tumors—one MMR-deficient ovarian cancer, one MMR-deficient endometrial cancer and one MMR-proficient CRC—from two carriers of *POLE* p.Met294Arg, were analyzed. All three displayed hyper/ultramutation (10.96–300.85 Mut/Mb). Signature 10 was identified in the CRC (contribution: 23%), and signature 14, linked to the co-occurrence of MMR deficiency and *POLE* ED pathogenic variant in the ovarian tumor (contribution: 15.32%). MMR-deficient associated signatures were the major contributors in the ovarian and endometrial tumors (Fig. 4; Supplementary Table S7).

While the breast tumor from the *POLE* p.Gly380Cys carrier showed no hypermutation (0.67 Mut/Mb), a breast tumor from a *POLE* p.Asp287Glu carrier, a melanoma from a *POLE* p.Ile307Val carrier, the MMR-proficient CRC from the *POLE* p.Ala426Val and *CHEK2* c.593–1G>T carrier, and a CRC from a *POLD1* p.Arg521Gln carrier showed hypermutation but none of the *POLE/D1*-associated mutational signatures (Fig. 4; Supplementary Table S7).

To help characterize the identified variants, we used the TCGA and COSMIC tumor sequencing data to see if those variants, when occurring somatically and in absence of additional somatic *POLE/D1* ED variants, caused a hyper/ultramutated phenotype and the *POLE/D1* ED-associated signatures. Considering the ED variants identified in the study, only two, *POLD1* p.Arg352Cys and p.Arg525Trp, fulfilled the abovementioned conditions. A CRC and a lymphoid neoplasm harboring *POLD1* p.Arg352Cys, as well as a stomach carcinoma with *POLD1* p.Arg525Trp, displayed hypermutation but not the *POLE/D1* ED-associated signatures (Supplementary Table S8). In the case of the CRC and the stomach carcinoma, the hypermutation detected was due to the presence of microsatellite instability (MSI) (MMR deficiency signatures’ contribution: 77%), and to the presence of *BRCA2* p.Pro1088Leufs*16 (*BRCA1/2* signature contribution [3]: 52%), respectively. In the *POLD1* p.Arg352Cys hematologic tumor, in absence of DNA repair alterations, no *POLE/D1* ED-associated signatures were identified.

### ED variant classification

Sixteen (16/2813; 0.57%) probands carried novel or rare ED missense variants. Taking into account several lines of evidence (functional data, tumor mutation burden and signatures, in silico predictors, cosegregation, and case–control data), and applying the ACMG/AMP guidelines for variant classification, *POLE* p.Met294Arg was classified as pathogenic; *POLE* p.Asp287Glu, *POLE* p.Asn336Ser, *POLD1* p.Arg352Cys, and *POLD1* p.Arg521Gln as likely benign; and *POLE* p.Ile307Val, p.Gly380Cys, p.Lys425Arg, and p.Ala426Val, and *POLD1* p.Ile307Met, p.Ile325Val, and p.Arg525Trp as variants of unknown significance (VUS) (Table 1). Details of the criteria considered for the classification of each variant are shown in Supplementary Table S3.

### Germline loss-of-function *POLE* and *POLD1* variants

Six LoF heterozygous variants were identified in the retrospective (*n* = 1) and prospective (*n* = 6) cohorts: *POLE* c.1185_1188delGGAG (p.Glu396Thrfs*15), c.4480C>T (p.Gln1494*), and c.2297_2298insA (p.Tyr766*); and *POLD1* c.230delC (p.Pro77Leufs*92), c.1195C>T (p.Gln399*), and c.3305delC (p.Pro1102Leufs*22) (Supplementary Table S9). The most prevalent phenotype in the carrier families was breast cancer (also the most represented tumor type in the studied cohorts), diagnosed in eight carriers (five families) (pedigrees: Supplementary Fig. S4). The *POLD1* p.Gln399* carrier also carried a *BRCA1* pathogenic variant (exon 8–13 deletion).

*POLE* c.1185_1188delGGAG (p.Glu396Thrfs*15), novel and disrupting the ED, perfectly segregated with breast cancer in the carrier family: the variant was present in the proband, her mother, and two maternal aunts, all four of whom were diagnosed with breast cancer (ages at diagnosis: 28, 71, 64, and 67, respectively). Two primary breast tumors from two carriers of *POLE* p.Glu396Thrfs*15 were analyzed by exome sequencing. None of the tumors showed hypermutation (1.09–1.70 Mut/Mb) or a *POLE*-associated signature (Fig. 4; Supplementary Table S7). Similar results were observed when analyzing two CRC tumors with a somatic variant in that same residue (Supplementary Table S8). No additional *POLE* or *POLD1* somatic pathogenic variants were detected in the tumors, discarding a somatic second hit.

Tumor exome sequencing performed in the MMR-deficient colon cancer developed by the *POLD1* c.230delC (p.Pro77Leufs*92) carrier revealed that ~85% of the signatures’ contribution corresponded to MMR deficiency and no representation of *POLE/D1*-associated signatures (Fig. 4; Supplementary Table S7). Sequencing data from a breast tumor harboring a somatic frameshift variant in the same residue as the germline *POLD1* c.3305delC (p.Pro1102Leufs*22) showed neither hypermutation nor the *POLE/D1*-associated mutational signature (Supplementary Table S8).

The frequency of LoF variants in familial cancer (and all the subtypes considered separately) is almost identical to that observed in cancer-free controls (0.22% [5/2309] in cases vs. 0.20% [119/59,095] in controls, *p* value = 0.81), thus agreeing with their lack of association with cancer predisposition^41^ (LoF variants present in the European population data set [gnomAD] are detailed in Supplementary Table S10). Moreover, tumor sequencing demonstrated no increased mutation burden, supporting intact DNA repair.

### Variants identified outside the ED

We identified 142 missense variants outside the ED (*POLE* *n* = 92 and *POLD1* *n* = 50) in 354 patients, with no relative overrepresentation among any of the phenotype-based groups. Four *POLD1* and 19 *POLE* outside-ED variants, identified in 29 individuals, were predicted deleterious (Supplementary Table S11).

Cosegregation data suggested that *POLE* c.671A>G (p.Tyr224Cys), the closest to the ED (44 amino acids upstream), was not associated with the increased breast cancer risk observed in the carrier family (Supplementary Table S11); observation supported by a yeast functional assay that revealed no mutator effect (Fig. 2). No cosegregation analyses could be performed for the remaining outside-ED predicted pathogenic variants. Tumors harboring the same somatic outside-ED missense variants in absence of other (likely) pathogenic *POLE* variants did not have ED-associated mutational signatures. Eight of the 23 predicted pathogenic variants (41/142 of all variants) could be analyzed in different tumor types with this approach (Supplementary Table S8). The frequency of outside-ED predicted pathogenic variants in familial cancer (and all the subtypes considered separately) does not differ from that observed in cancer-free controls (1.26% [29/2309] in cases vs. 0.91% [539/59,095] in controls, *p* value = 0.10) (Supplementary Table S12).

### Tumor spectrum and prevalence of germline *POLE* and *POLD1* ED variants in hereditary cancer

Two hereditary cancer families (2/2813; 0.07%) carried a pathogenic variant in *POLE* (p.Met294Arg), making a total of 5 carriers. The phenotypic spectrum of the carriers included (1) CRC in absence of adenomatous polyposis, diagnosed in 4 of the 5 carriers (80%) (mean age at CRC diagnosis 51; range 37–57), one of whom had developed three metachronous colorectal tumors; (2) endometrial cancer, diagnosed in two carriers (40%) (mean age at diagnosis 46; range 36–55); and (3) ovarian cancer, diagnosed in one carrier (20%) (age at diagnosis 36). Three ED variant carriers (60%) developed ≥2 primary tumors. The prevalence of (likely) pathogenic ED variants, being of 0.09% (2/2309) in the prospective familial cancer cohort, reached 0.33% (2/601) when restricted to CRC and polyposis phenotypes (Fig. 1; Supplementary Table S13).

The frequency of rare ED missense variants—including pathogenic and VUS—in *POLE* and *POLD1* in hereditary cancer was 0.39% (9/2309). Almost half of the pathogenic variants and VUS (4/9; 44.44%) were found in patients with a referral of hereditary nonpolyposis CRC or polyposis, who accounted for 26% (601/2309) of the total number of patients tested. In this group of patients, the prevalence of ED variants increased to 0.67%. With regard to the most represented group of patients, i.e., those referred for genetic testing as hereditary breast and/or ovarian cancer, the frequency of ED variants was 0.27% (4/1468) (Fig. 1; Supplementary Table S13).

## DISCUSSION

*POLE* and *POLD1* ED variant interpretation is challenging and the inclusion of multiple pieces of evidence is highly advisable. Population and cosegregation data, in silico prediction of pathogenicity, location within the DNA binding cleft, results of the functional repair assay, and tumor mutational data were taken into consideration for variant classification. Based the relevance of each piece of evidence, we adapted the ACMG/AMP variant classification guidelines to the particular conditions of *POLE* and *POLD1* ED variants and to the relevance of the functional information gathered, i.e., tumor mutational signatures, yeast repair assay results, and alteration of the DNA binding pocket (Table S3).

We consider tumor sequence analysis of utmost importance for a proper classification of *POLE* and *POLD1* ED variants. Mutational signature 10, in the presence of hyper/ultramutation, is unequivocally linked to the presence of an ED pathogenic variant—a priori independently of the tumor type analyzed—however, one must be certain of the inexistence of a somatic ED pathogenic variant that could be causing the presence of the *POLE/D1*-associated tumor molecular phenotype. Moreover, a challenging situation occurs when the tumor we analyze has other DNA repair defects, e.g., MMR deficiency (MSI). In this situation, if the mutational signature analysis does not reveal any of the *POLE/D1*-associated signatures, i.e., signatures 14 or 20 when the germline variant affects the ED of *POLE* or ED respectively, it is highly advisable to analyze a second tumor, ideally MMR proficient, developed in the same family or harboring the same variant but in a somatic context, verifying that no other somatic ED variant is present.

Due to the high homology of the ED of *POLE* and *POLD1* in human and yeast, this model has been widely used for variant functional assessment. In our study, unequivocal repair impairment (*p* < 0.001) was observed for *POLE* p.Leu424Val (positive control), p.Met294Arg (likely pathogenic), and p.Ala426Val (uncertain significance), but also for p.Asp287Glu, a variant classified as likely benign based on cosegregation data, frequency in cases and controls, and absence of an ED-associated mutational signature. It seems evident that, in addition to the (likely) pathogenic variants directly affecting the exonuclease catalytic residues, such as *POLD1* p.Asp316Gly, p.Asp316His, ED variants affecting residues in direct contact or in very close proximity to the DNA fragment, i.e., in the DNA binding pocket, have a very high chance of being pathogenic. In fact, the vast majority of *POLE* and *POLD1* (likely) pathogenic variants reported to date are localized in the DNA binding cleft, including *POLE* p.Thr278Lys, p.Met294Arg (this study), p.P286L, p.N363K, p. D368N, p.Leu424Val, and p.Pro436Ser, as well as *POLD1* p.Leu474Pro and p.Ser478Asn (Supplementary Table S2). Hamzaoui et al. observed that variants interfering with DNA binding (p.P286L and p.N363K) produce a higher mutagenic effect in yeast than variants disrupting ion metal coordination at the exonuclease site.^35^ In line with Barbari et al.,^15,40^ our results indicate that any variant located in the DNA binding cleft shows a mutator effect in yeast, even if it is not pathogenic, as occurs with p.Asp287Glu. Based on this and the high variability of the yeast assay among replicates and experiments, also observed in other studies,^35,40^ we have used its results with caution. We posit that yeast assay observations may be considered supporting or, at most, moderate (not strong) evidence for ACMG/AMP variant classification, depending on the level of significance when compared with the wildtype clone.

In our study, two families carrying *POLE* and *POLD1* VUS also carried pathogenic variants in *ATM* and *CHEK2*, two genes that confer a moderate risk of breast cancer.^37,38^ The aggressive phenotype developed by the *POLD1* p.Arg525Arg and *ATM* (p.Ser2407*) carrier supports a cumulative effect of the two variants, while that may not be the case for the carriers of *POLE* p.Ala426Val (VUS) and *CHEK2* c.593–1G>T, where two of the three carriers developed late-onset CRC. Hamzaoui et al. identified two relatives who carried both *POLE* p.K425R (VUS) and *MSH2* c.942+3A>T (pathogenic), in a family with multiple CRC-affected members diagnosed at extremely young ages (range: 19–33), suggesting a cumulative effect of the two variants.^35^ Whether the aggregation of cancer in these families is explained only by the pathogenic variants in *ATM*, *CHEK2*, *or MSH2*, or the combined effect with the *POLE/D1* variants (all of them classified as VUS until now), remains to be elucidated.

The role of LoF and outside-ED *POLE* and *POLD1* heterozygous variants has been a matter of controversy since the description of PPAP in 2013. Our data suggest that these variants are most likely nonpathogenic, at least not associated with PPAP—this being relevant to avoid misdiagnoses in the clinical setting. A particular case is that of *POLE* p.Glu396Thrfs*15, identified in four breast cancer–affected women of the same family in absence of additional pathogenic variants in known or candidate hereditary cancer genes (exome sequencing; data not shown). Despite the absence of tumor hypermutation and *POLE-*associated mutational signatures, whether the variant is the cause of the cancer aggregation in the family remains to be elucidated, and if so, what is the molecular mechanism underlying its potential causal effect.

In conclusion, our findings indicate that PPAP constitutes 0.1–0.4% of familial cancer cases referred to a hereditary cancer clinic, 0.3–0.7% when considering nonpolyposis CRC and polyposis patients, with the most commonly associated tumor types being colorectal, endometrial, and ovarian cancers. ED variant interpretation is challenging and should include multiple pieces of evidence. LoF and outside-ED variants are not associated with PPAP, a cancer-predisposing syndrome characterized by a defect in the proofreading activity of polymerases epsilon and delta.

## Supplementary information

Supplementary Material

Supplementary Table S3

Supplementary Table S4

Supplementary Table S7

Supplementary Table S8

Supplementary Table S10

Supplementary Table S11

Supplementary Table S12
