# Supplementary material for: Role of POLE and POLD1 in familial cancer
Source: Genet Med. 2020 Aug 14;22(12):2089–100. doi: 10.1038/s41436-020-0922-2 (PMC7708298; doi:10.1038/s41436-020-0922-2)
Supplement: Supplementary file 1 — Supplementary Material [file 41436_2020_922_MOESM1_ESM.pdf]

SUPPLEMENTARY MATERIAL

Supplementary Materials and Methods

DNA and RNA extractions

Genomic DNA from peripheral blood was extracted using the FlexiGene DNA kit (Qiagen), and from formalin-fixed paraffin-embedded samples, using the QIAamp DNA FFPE tissue Kit (Qiagen, Hilden, Germany), following the manufacturer's instructions. RNA from cultured lymphocytes was extracted using a standard Trizol-based protocol.

DNA mismatch repair (MMR) protein expression and microsatellite instability

Staining of MMR proteins in paraffin-embedded tumor tissue was performed using the BenchMark XT automated tissue staining system (Ventana Medical Systems, Inc., Tucson, AZ). Microsatellite instability (MSI) was assessed with the Microsatellite Instability Analysis Kit (Promega, Madison, WI).

Variant nomenclature

Variant nomenclature is according to HGVS recommendations (version 19.01) with nucleotide 1 corresponding to the A of the ATG translation initiation codon.

Yeast assay

The pFA6α-KanMX6 vector was used to clone wild-type *pol2* gene from *Schizosaccharomyces pombe* (yeast homolog of human *POLE*), as previously described.[1] Human *POLE* variants that were conserved in *S. pombe* (p.D287E, p.M294R, p.I307V, p.G380C, p.A426V, p.Y224C, and p.L424V) were generated by the QuikChange II XL Site-Directed Mutagenesis Kit (Stratagene, La Jolla, CA), according to manufacturer's conditions. Sanger sequencing was used to verify the presence of the variants. Primer sequences are detailed in Supplementary Table S14.

Adenine-defective (ade6-485) *S. pombe* was transfected with the linearized plasmid carrying *pol2* wildtype, the ED mutation-positive control *pol2-L425V* (≡*POLE* p.L424V), or the identified variants, and grown in adenine-deficient media. Two independent colonies were used for each construct. A total of $1x10^7$cells/plate was set up for exonuclease repair ability assay, and experiments were performed in triplicate. Mutation rates were calculated after 12 days comparing the *ade6*-485 allele reversion rate of the *pol2* wildtype (negative control) with the ED mutation-positive control and the corresponding variants. Mann-Whitney non-parametric test was used to compare *ade6*-485 allele reversion rate of the *pol2* wildtype (negative control) with the ED mutation-positive control (*pol2*-L425V), and the corresponding variants. Graphs and associated statistical analysis were performed using GraphPad Prism version 5 for Windows (GraphPad Software, San Diego, CA).

Tumor mutational analysis

Whole exome sequencing of DNA extracted from the tumors and the corresponding blood samples was carried out in a Hi-Seq2000 (Illumina) with a mean coverage per sample of >70x for FFPE and >60X for fresh tumor tissue, after library preparation using the Agilent Sure Select Human All Exon v5 kit. Sequencing was performed at Centro Nacional de Análisis Genómico (CNAG, Barcelona, Spain). After quality control assessment using FastQC software, WES raw data were pre-processed with Trim Galore! (v0.4.0) for adapters and bad quality reads removal. Sequence alignment against reference human genome (hg19/GRCh37) was carried out with BWA (v.0.7.15)[2] and alignments were processed for duplicates' removal and base recalibration according to GATK best practices (https://gatk.broadinstitute.org). Variants were called with MuTect2 following the GATK4 pipeline.[3] Variants identified in the patient's blood DNA were eliminated for the analysis of somatic mutations in the tumor. Variants present in at least 10 reads, minimum Phred quality of 20, and minimum 10% of tumor allele frequency, were considered for subsequent analyses. The contribution of COSMIC mutational signatures[4,5] was calculated with DeconstructSigs[6] using the R package. Total mutation burden was estimated by considering single nucleotide variants (SNV) from exonic regions. Tumor samples developed by carriers of the pathogenic *POLE* p.L424V and *POLD1* p.D316G and p.D316H variants, previously identified by our group,[7,8] were used as ED mutation-positive controls. The absence of somatic *POLE* and *POLD1* mutations was confirmed in all tumors. TCGA and COSMIC tumor sequencing data from samples harboring somatic *POLE/POLD1* ED variants and affecting the same amino acids as the germline variants identified in this study, were also analyzed following the same workflow.

Case-control study

The frequencies of rare variants in *POLE* and *POLD1* were assessed in a population-based multi case-control study (MCC-Spain,[9] https://www.mccspain.org/). Cases and controls, all of them from Spain, had been genotyped with the Illumina Infinium Human Exome Bead Chip array, which includes rare variants in coding regions identified in the 1000 Genomes Project. For the purpose of our study, we analyzed genotyping data from the CRC case-control series, which includes 1,336 CRC patients and 2,744 cancer-free controls, and from the breast cancer case-control study, which includes 1,138 breast cancer patients and 1,240 controls. Fisher's exact test was used to compare genotype frequencies between cases and controls.

Classification of germline *POLE* and *POLD1* exonuclease domain missense mutations

The following specific pieces of evidence were incorporated to the ACMG/AMP guidelines for the classification of *POLE* and *POLD1* ED variants (shown in detailed in Supplementary Table S3). PM1 was used if the affected residue is located at the exonuclease domain and within the DNA binding cleft. REVEL *in silico* modeling program was used to predict pathogenicity, being PP3 evidence applied when REVEL score was ≥0.35, and BP4 if REVEL score <0.30. PS3_supporting or moderate was applied, when a mutator phenotype in *S. pombe* was observed at moderate (++, p=0.01-0.001) or high levels (+++, p<0.001), respectively. BS3_supporting was considered when no mutator effect was observed in yeast (p>0.05). For cases with WES data available from

a carrier's tumor, mutation burden and mutational signature was applied as PP4 (following the recommendations from Walsh et al.[10]), always in absence of a somatic ED (suspected) pathogenic variant. For variants without sequencing data from carriers' tumors, TCGA/COSMIC sequencing data from tumors harboring the same ED variant was used to obtain mutation burden and signature data, also making sure that no other *POLE/D1* ED (suspected) pathogenic variant was present in the analyzed tumor. PP4_strong was applied when the tumor showed hyper/ultramutation and *POLE/D1*-associated mutational signature 10. PP4_moderate was applied for MSI tumors with hyper/ultramutation and *POLE/D1*-associated mutational signatures 14 or 20 (depending on the affected polymerase). In contrast, benign evidence BP5 (variant found in a case with an alternate molecular basis for disease) was considered when an MMR-proficient tumor was not hyper/ultramutated or when a DNA repair-proficient tumor was hypermutated but did not display mutational signatures 10, 14 or 20. This BP5 was directly applied when a tumor harboring the somatic variant was analyzed, however, when the analysis involved tumor(s) developed by germline variant carriers, BP5 was applied when the conditions were fulfilled by at least two tumors, in order to minimize the possibility of a false classification due to the presence of a phenocopy. Additional supporting benign evidence BS1 (allele frequency greater than expected for disorder) was considered if MAF>0.1% in any gnomAD v2.1.1 non-cancer sub-population, and BS2_supporting, when the variant was observed in ≥10 healthy adult individuals (above 60 years of age) for a dominant heterozygous disorder (not applied when BS1 was considered).

Supplementary Table S1. Phenotypic description of the cohorts included in the study. None of the patients carried germline pathogenic variants in the known high-penetrance cancer genes associated with the patient and/or family's phenotype; i.e., breast and/or ovarian cancer patients had no germline pathogenic variants in *BRCA1* or *BRCA2*; CRC or associated tumors (endometrial, gastric, small intestine) were MMR proficient (no Lynch syndrome); and Li-Fraumeni-suspected patients did not carry germline pathogenic variants in *TP53*.

RETROSPECTIVE COHORT (n=504):

**Selection criteria**	**# of patients**
Personal and/or familial breast and ovarian cancer history	192
[a]Personal and/or family history of: CRC, endometrial, small intestine or gastric cancer *&* breast or ovarian cancer	122
Personal and/or family history of: Brain cancer & any other tumor	22
[b]Personal and/or family history of: Breast, endometrial, brain or skin cancer & >5 polyps	34
Personal and/or family history of: Multiple primary tumors (excluding the tumor combinations included in the other categories)	30
Patients/families with no germline *TP53* mutation fulfilling the following criteria[11]<br>Classic Li-Fraumeni (n=3)<br>Li-Fraumeni-like (n=15)<br>Chompret / Revised Chompret (n=61)<br>Eeles (n=25)	104
**Total**	**504**

PROSPECTIVE COHORT (n=2,309):

**Clinical phenotypes**	**# of patients**
Personal and/or familial breast and ovarian cancer history	267
Personal and/or family history of: Ovarian cancer	317
Personal and/or family history of: Breast cancer	884
Hereditary non-polyposis CRC (HNPCC)	354
Familial adenomatous polyposis (FAP/AFAP)	247
Li-Fraumeni	15
Other tumors:<br>Melanoma, prostate, pancreas, among others.	225
**Total**	**2,309**

[a]Families/patients fulfilling the classical criteria for HNPCC (Amsterdam or Bethesda) were not included in this cohort.
[b]The polyps were either adenomas or hyperplastic polyps. Classic/attenuated colonic adenomatous polyposis had been previously studied (Bellido *et al.*[7]) and were not included in this study.

Supplementary Table S2. *POLE* and *POLD1* germline variants classified as (likely) pathogenic with sufficient supporting evidence in the literature and their localization from the ssDNA substrate. Structural localization was performed using the *S. cerevisiae* DNA polymerase ε (PDB: 4M8O), DNA polymerase δ (PDB: 3IAY) and the single-stranded DNA from the bacteriophage T4 polymerase complex (PDB: 1NOY). All variants are localized in the DNA-binding cavity (at least one atom of these residues is accessible in the cavity where the DNA binds) according to the CASTp method (http://sts.bioe.uic.edu/castp/calculation.html). Exo motifs previously defined by Shevelev et al.[12]

***POLE/POLD1* variant**	**DNA binding cavity**	**Exo Motif location**	**REVEL score**	**Source**
POLD1 c.1421T>C (p.Leu474Pro)	Yes	EXO IV	0.913	Valle *et al.*[8]
POLD1 c.1433G>A (p.Ser478Asn)	Yes	EXO IV	0.377	Palles *et al.*[10]
POLD1 c.947A>G (p.Asp316Gly)	Yes	EXO I (catalytic residue)	0.773	Bellido *et al.*[7]
POLD1 c.946G>C (p.Asp316His)	Yes	EXO I (catalytic residue)	0.743	Bellido *et al*.[7]
POLE c.833C>A (p.Thr278Lys)	Yes	EXO I	0.666	Castellsagué *et al.*[13]

POLE/POLD1 variant	DNA binding cavity	Exo Motif location	REVEL score	Source
POLE c.857C>T (p.Pro286Leu)	Yes	close to EXO I (a.a. 271-285)	0.812	Hamzaoui *et al.*[14]
POLE c.c.881T>G (p.Met294Arg)	Yes	close to EXO I (a.a. 271-285)	0.815	This study / Hamzaoui *et al.*[14]
POLE c.1089C>G (p.Asn363Lys)	Yes	EXO II	0.735	Rohlin *et al.*[15] / Vande Perre *et al.*[16]/ Hamzaoui *et al.*[14]
POLE c.1102G>A (p.Asp368Asn)	Yes	EXO II	0.529	Hamzaoui *et al.*[14]
POLE c.1270C>G (p.Leu424Val)	Yes	EXO IV	0.654	Palles *et al.*[17] / Valle *et al.*[8]/ Hamzaoui *et al.*[14]
POLE c.1306C>T (p.Pro436Ser)	Yes	EXO V	0.524	Spier *et al.*[18] / Hamzaoui *et al.*[14]
POLE:c.1231G>C (p.Val411Leu)	Yes	Flanking EXO IV (in alpha-helix defining DNA binding pocket)	0.457	Wimmer *et al.*[19]

Supplementary Table S3. Description of evidence used for *POLE/D1* ED missense variant classification according to the ACMG/AMP guidelines.

[Included as separate Excel file]

Supplementary Table S4. Frequency of germline (L)P variants in cancer predisposing genes identified in the prospective cohort (2,309 unrelated hereditary cancer patients). Results from a hereditary cancer gene panel.[20,21]

[Included as separate Excel file]

Supplementary Table S5. Clinical characteristics of *POLE* c.861T>A (p.D287E) carrier families identified in this study and others previously reported.

Carrier family (reference)	# carriers / total cancer affected individuals	Carriers' phenotypes (age at diagnosis)	Non-carriers' phenotypes (age at diagnosis)	Other cancers in the family
1 (Aoude et al.)[22]	2/6	1. Melanoma (70) and non-Hodking lymphoma (40)<br>2. Melanoma x8 (47) and Breast ca. (78) and SCC (73)	Melanoma (22); Melanoma (30); Melanoma (32); Melanoma (34)	Ewing's sarcoma (14)
2 (Aoude et al.)[22]	1/2	Melanoma (40)	Melanoma (40)	No
3 (Aoude et al.)[22]	1/1	Melanoma (57)	n.a.	Pharynx SCC (77)
4 (Jansen et al.)[23]	1/1	[a]CRC (53)	n.a.	No
5 (current study)	1/2	Breast ca. (40)	Ovarian ca. (53)	No
6 (current study)	1/1	Breast ca. (59) and CRC (59)	n.a.	CRC (87); Endometrial ca. (58); Pancreatic ca. (59)

[a]The tumor harbored two somatic *MLH1* pathogenic variants: c.208-1G>A; p.? and c.440_447del; p.(G147Dfs*22).
Abbreviations: ca., cancer; CRC, colorectal cancer; SCC, squamous cell carcinoma.

Supplementary Table S6. Allele frequency of *POLE* c.861T>A (p.D287E) assessed in cancer cases and controls from European populations, including data from the MCC_Spain case-control study, Collaborative Spanish Variant Server (CSVS), and the current study.

	[a]MCC_Spain				[b]CSVS	Current study
POLE/D1* variant *(rs ID)	**CRC patients**	**Controls**	**Breast cancer patients**	**Controls**	**Spanish controls**	**Prospective hereditary cancer cohort**
POLE c.861T>A; p.D287E (rs139075637)	1/2,672 (0.04%)	1/5,488 (0.02%)	0/2,276 (0.00%)	1/2,480 (0.04%)	1/3,101 (0.03%)	2/4,618 (0.04%)

[a]Exome array data from MCC Spain which includes 1,348 CRC patients and 2,744 controls; and 1,138 breast cancer patients and 1,240 controls (https://shiny.snpstats.net.exome/). Data included correspond to actual genotyping results (not imputed).
[b]Collaborative Spanish Variant Server (http://csvs.babelomics.org/); non-cancer individuals.

Supplementary Table S7. Mutational signature contribution (DeconstructSigs) in tumors developed by carriers of *POLE/POLD1* germline ED or LoF variants.

[Included as separate Excel file]

Supplementary Table S8. Mutational burden and mutational signature contribution in tumors harboring somatic *POLE/POLD1* (ED, outside-ED and LoF) variants identical to the germline variants identified in the current study. In bold, hyper/ultra-mutated samples, somatic *POLE/POLD1* pathogenic variants, and *POLE/POLD1*-associated mutational signature. Outside-ED predicted pathogenic variants are shaded in grey. Somatic variants and sequence data were extracted from COSMIC and TCGA databases.

[Included as separate Excel file]

Supplementary Table S9. Loss-of-function (LoF) *POLE* and *POLD1* germline variants identified in the current study (population MAF<1%).

[a]Genetic variant and domain (Exon #) (Suppl. Figure S1)	dbSNP (rs#) Population MAF % (GnomAD NFE)	[b]Evolutionary conservation (PhyloP/ PhastCons)	Proband phenotype (see Suppl. Figure S4)	Familial cancer history and cosegregation results (see Suppl. Figure S4)	Tumor sequence analysis (Mutational burden and signature contribution) (see Figure 3 and Suppl. Table S6)	COSMIC/TCGA tumor sequence analysis (Mutational burden and signature contribution) (see Suppl. Table S7)
POLE						
c.1185_1188delGGAG; p.Glu396Thrfs*15 Exonuclease (12)	n.a.	n.a.	**Fam Q:** BC 28	Mother (BC 71), two aunts (BC 64 and 67) *Carriers:* All BC affected	**I.2 (BC):** 1.1 Mut/Mb **I.3 (BC):** 1.7 Mut/Mb	p.396fs*16: **TCGA (CRC-1):** 69.74 Mut/Mb; 36.8% MMR-d sigs **COSMIC (CRC-2):** 102.82 Mut/Mb; 73.7% MMR-d sigs
c.2297_2298insA; p.Tyr766* Outside (20)	n.a.	Non-conserved (-0.455/0.032)	**Fam R:** BC 43	No familial cancer history	n.a.	No
c.4480C>T; p.Gln1494* Outside (35)	n.a.	Conserved (8.076/1)	**FamS:** BC 32 and Thyroid ca. 28	Aunt (BC 55), grandmother (BC 78 and stomach ca. 88) *Carriers:* n.a.	n.a.	No
POLD1						
c.230delC; p.Pro77Leufs*92 Outside (3)	n.a.	n.a.	**Fam T:** CRC 40	Mother (BC, 59 and HP 60), grandfather (CRC and polyps 66) *Carriers:* n.a.	**III.2(CRC):** 37.4 Mut/Mb; 84% MMR-d sigs	No
c.1195C>T; p.Gln399* Exonuclease (10)	n.a.	Conserved (6.550/1)	**Fam U:** BC 49 and LC 56	No familial cancer history	n.a.	No
c.3305delC; p.Pro1102Leufs*22 Outside (27)	rs761614971 (0.0016)	n.a.	**Fam V:** BC 39	No familial cancer history	n.a.	p.P1102fs*6: **COSMIC (Esophagus ca.):** 7.71 Mut/Mb; no *POLE/D1*, no MMR-def sigs **TCGA (BC):** 1.73 Mut/Mb; no POLE/D1, no MMR-def sigs

Abbreviations: BC, breast cancer; ca., cancer; CRC, colorectal cancer; Eur-NF, European population (Non-Finnish); GnomAD, Genome Aggregation Database; HP, Hyperplastic polyposis; LC, lung cancer; MAF, minor allele frequency; MMR-d., mismatch repair deficiency; n.a., not available information; Signat., Mutational Signature.

[a]RefSeq GRCh37: *POLE* (NM_006231.2; NP_006222.2) and *POLD1* (NM_001256849.1; NP_001243778.1).
[b]PhyloP/PhastCons values were obtained from alignments of 100 vertebrate genome sequences. The higher the score, the more conserved the site (PhyloP score: -20 to +10, PhastCons: 0 to 1).

Supplementary Table S10. *POLE* and *POLD1* LoF variants (considering the canonical transcript) in control population (gnomAD v.2.1.1 non-Finnish, non-cancer Europeans; consultation date: March 2020).

[Included as separate Excel file]

Supplementary Table S11. *In silico* prediction of pathogenicity and clinical features of the carriers of variants located outside the exonuclease domain.

[Included as separate Excel file]

Supplementary Table S12. *POLE* and *POLD1* outside exonuclease domain variants (considering the canonical transcript) in control population (gnomAD v.2.1.1 non-Finnish, non-cancer Europeans; consultation date: March 2020).

[Included as separate Excel file]

Supplementary Table S13. Prevalence of *POLE* and *POLD1* germline variants in hereditary cancer using data from the prospective cohort (2,309 hereditary cancer unrelated patients).

**Type of *POLE/D1* variants**	**Hereditary cancer N (%)**	**HNPCC N (%)**	**Polyposis N (%)**	**HBOC, BrCa, OvCa N (%)**	**Others N (%)**
Total # of individuals	**N=2,309**	N=354	N=247	N=1,468	N=225
**[1]ED**	**9 (0.39%)**	3 (0.85%)	1 (0.40%)	4 (0.27%)	1 (0.45%)
**[2]LoF**	**5 (0.22%)**	1 (0.28%)	0 (0.00%)	4 (0.27%)	0 (0.00%)
**[3]Outside-ED**	**29 (1.26%)**	4 (1.13%)	4 (1.62%)	18 (1.23%)	3 (1.33%)

Abbreviations: BrCa, Breast cancer; ED, exonuclease domain; HBOC, Hereditary breast and ovarian cancer; LoF, Loss-of-function; OvCa, ovarian cancer.

[1]Exonuclease domain variants classified as variants of unknown significance or (likely) pathogenic by using the ACMG/AMP guidelines (manual curation).
[2]Frameshift, stop-gained, and start-lost variants were considered.
[3]Missense variants located outside the exonuclease domain and predicted pathogenic by REVEL (score ≥0.35).
[4]Source: gnomAD v.2.1.1 non-Finnish, non-cancer Europeans; considering only canonical transcript variants (consultation date: March 2020).

Supplementary Table S14. Primers used for cloning and sequencing in yeast, mutagenesis and cosegregation studies.

Name	Forward primer (5'-3')	Reverse primer (5'-3')	Size (bp)
**Cloning**			
Pol2 BglII-AscI	CGCGCGAGATCTCTGATTCGTTTAAGCTTTTTCAGTT AATGGTGG	CTAAAGGCGCGCCGGTACATTGAGGCGACATCAAGA TGG	2593
**Sequencing**			
Pol2 (hPOLE)	GACCTTCAGGCTGTCAGGAA	GCAACAGCATCTGAAACTGAA	881
**Mutagenesis**			
POLE p.Y224C	ATGATACGGAACATCGCACTCACGGATATCTAAAACA TGATTTAAAGG	CCTTTAAATCATGTTTTAGATATCCGTGAGTGCGATGT TCCGTATCAT	
POLE p.D287E	ATCGAACGAGCTTTCAGGAAACTTCAATGGCAGCTT	AAGCTGCCATTGAAGTTTCCTGAAAGCTCGTTCGAT	
POLE p.I307V	GGAAATAATTTCTCTATTGGTAACTAAAAATCCTTGG CCATCAATCATATAAG	CTTATATGATTGATGGCCAAGGATTTTTAGTTACCAAT AGAGAAATTATTTCC	
POLE p.A426V	CTAATTTACTGACAGTGACAACTTTGAGACCTTGACT TCCT	AGGAAGTCAAGGTCTCAAAGTTGTCACTGTCAGTAAA TTAG	
POLE p.L424V (C+)	GGGTAAAGAGAGATAGTTATTTACCTCAAGGAAGTC AAGGTGTCAAAGCTGTCACTGTCAGTAAATTAGGTTA TAATCC	GGATTATAACCTAATTTACTGACAGTGACAGCTTTGAC ACCTTGACTTCCTTGAGGTAAATAACTATCTCTCTTTA CCC	
POLE p.G380C	CGCACGTGCTGCATTTCACTGTTTGAATTTAACAGAA GAA	TTCTTCTGTTAAATTCAAACAGTGAAATGCAGCACGTG CG	
POLE p.M294R	GCTCGTTCGATAAAATAAGGATGATATCTTATATG	CATATAAGATATCATCCTTATTTTATCGAACGAGC	
**Cosegregation**			
POLE Exon 9	GGTGTTCAGGGAGGCCTAAT	TGCTGCTGTAGTATGGGGAC	242
POLE Exon 10	AGCCTCTGACTTGTGCTGAT	CACATGTCCGTTCTTCCCAC	205
POLE Exon 12	AGGAATGGAGAAAGGGGCAT	AAGAGGCCTTCAGATCTCGC	250

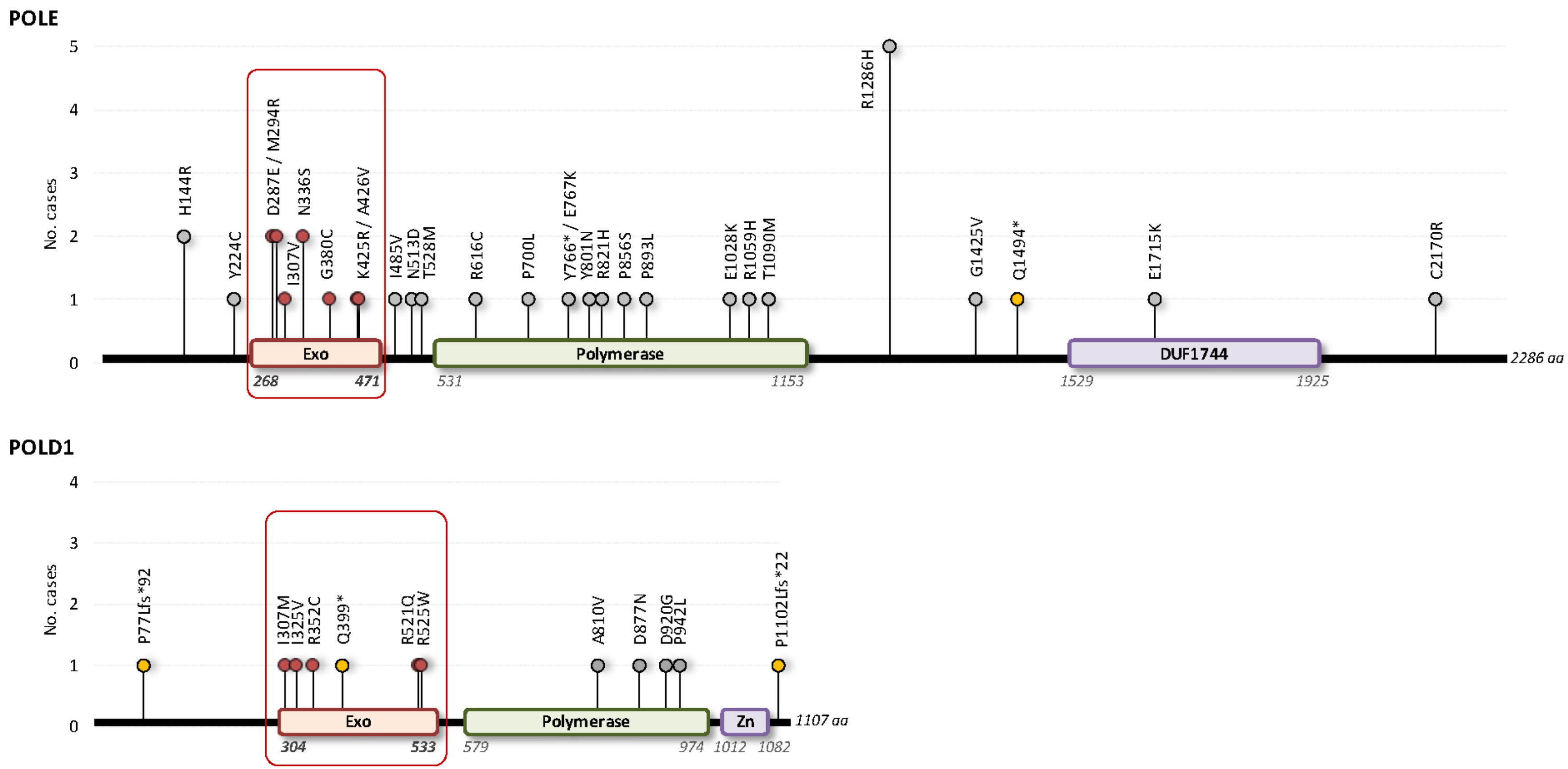

Supplementary Figure S1. Schematic representation of the domains of human *POLE* and *POLD1*, and location of ED missense, loss-of-function, and outside-ED predicted-pathogenic (REVEL≥0.35) variants identified in the prospective cohort. Y-axis represents the number of carrier families identified for each variant. X-axis shows the location of protein domains according to NCBI information (hPOLE: aa.268-471, 3'-5' exonuclease domain (exo); aa.531-1153, DNA polymerase type-B epsilon subfamily catalytic domain (pol); aa.1529-1925, domain of unknown function (DUF1744). hPOLD1: aa.304-533, 3'-5' exonuclease domain (exo); aa.579-974, DNA polymerase type-B delta subfamily catalytic domain (polymerase); aa.1012-1082, C4-type zinc-finger (Zn)). Missense variants located within the ED are highlighted in red, loss-of-function variants in orange, and missense variants outside the ED in grey.

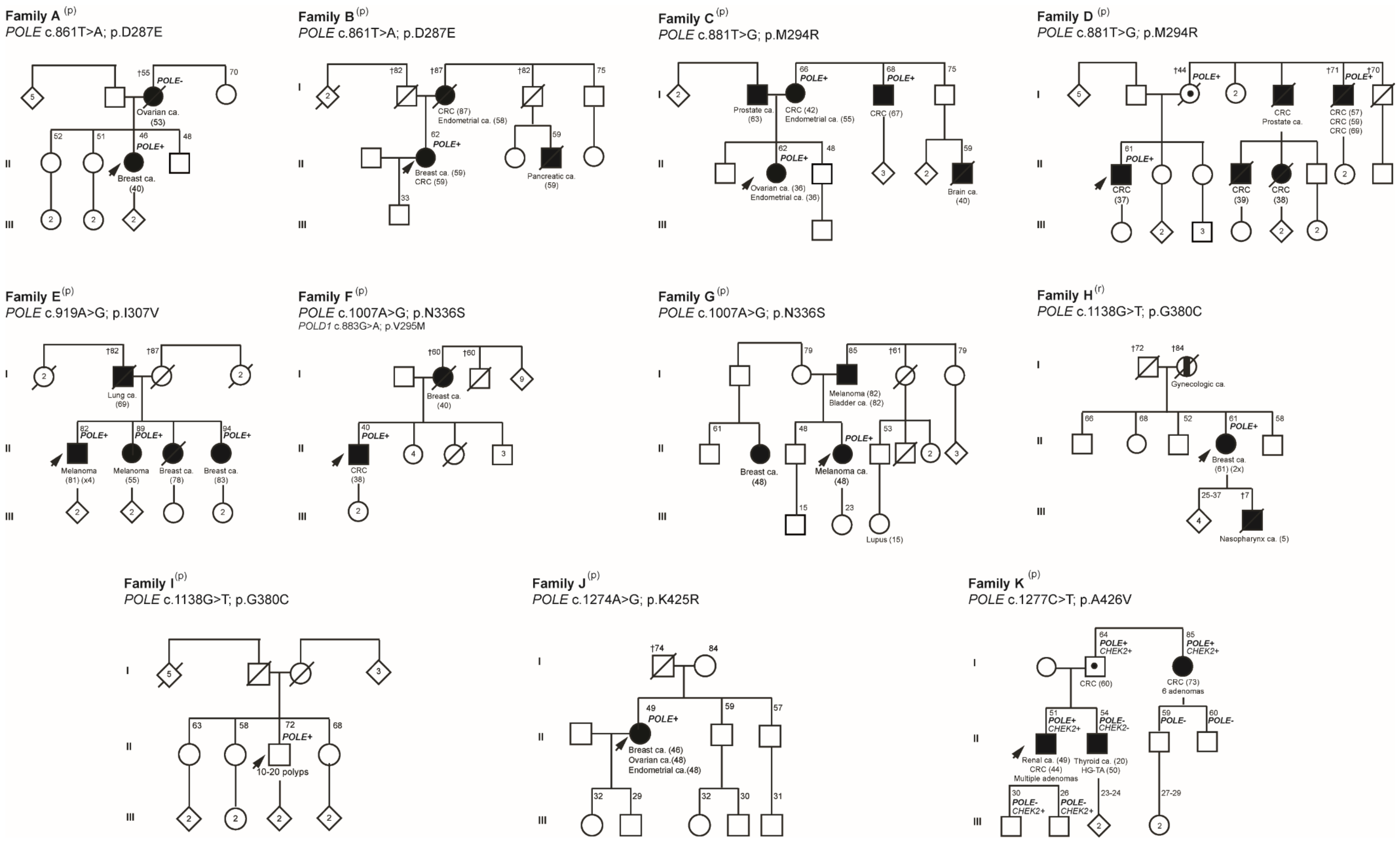

Supplementary Figure S2. Pedigrees of the families carrying *POLE* exonuclease domain germline variants. Filled symbol, cancer. Black point, obligate carrier. Black bar, diagnosis not confirmed. Ages at information gathering or at death (†), when available, are indicated on the top corner. The black arrow points out the index case. *POLE+*, *POLE* variant carrier; *POLE*-, non-carrier; *CHEK2+*, *CHEK2* c.593-1G>T carrier; *CHEK2*-, non-carrier. *Abbreviations:* ca., cancer; CRC, colorectal cancer; (p), family from the prospective cohort; (r), family from the retrospective cohort; HG-TA, tubular colorectal adenoma with high-grade dysplasia.

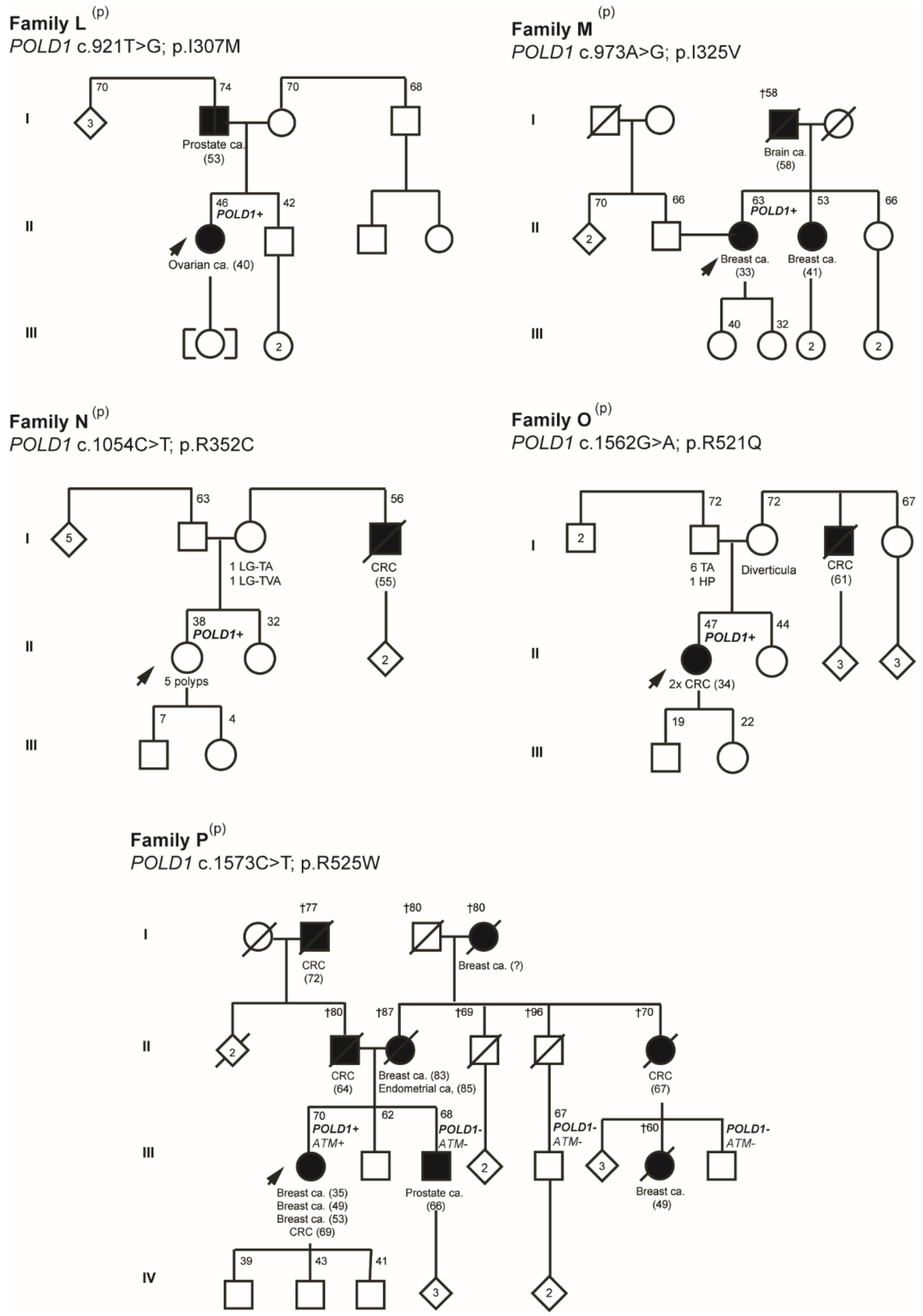

Supplementary Figure S3. Pedigrees of the families carrying *POLD1* exonuclease domain germline variants. Filled symbol, cancer. Ages at information gathering or at death (†), when available, are indicated on the top corner. The black arrow points out the index case. *POLD1+*, *POLD1* variant carrier; *POLD1-*, non-carrier of the *POLD1* variant identified in the family; ATM+, c.7220C>A (p.Ser2407*) carrier; ATM-, non-carrier of the *ATM* pathogenic mutation. *Abbreviations:* ca., cancer; CRC, colorectal cancer; (p), family studied in the prospective cohort; (r), family selected in the retrospective cohort; LG-TA, tubular colorectal adenoma with low-grade dysplasia; LG-TVA, tubulovillous colorectal adenoma with low-grade dysplasia.

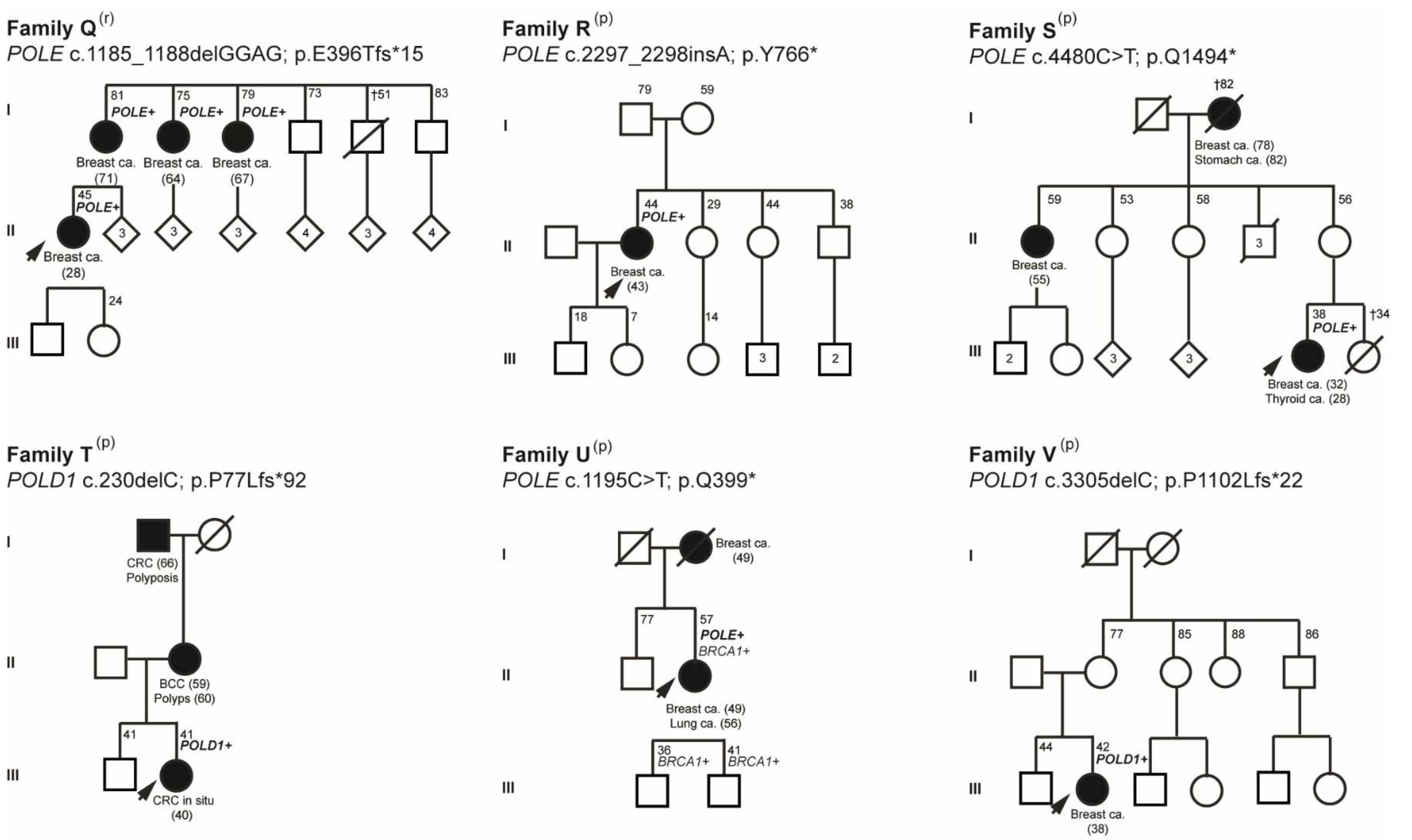

Supplementary Figure S4. Family pedigrees of the carriers of loss-of-function *POLE/POLD1* variants. Filled black symbol, cancer affected. Black bar, diagnostic not confirmed. Ages at information gathering or at death (†), when available, are indicated on the top corner, and ages at cancer diagnosis, after tumor type. Black arrow, index case; *POLE/D1+*, carrier of the variant identified in the family; *BRCA1+*, carriers of *BRCA1* exon 8 to exon 13 deletion. *Abbreviations:* BCC, basal cell carcinoma; ca, cancer; CRC, colorectal cancer; (p), family included in the prospective cohort; (r), family included in the retrospective cohort.

REFERENCES

1. Esteban-Jurado C, Giménez-Zaragoza D, Muñoz J, et al. POLE and POLD1 screening in 155 patients with multiple polyps and early-onset colorectal cancer. Oncotarget. 2017;8(16):26732-26743.
2. Li H, Handsaker B, Wysoker A, et al. The Sequence Alignment/Map format and SAMtools. Bioinformatics. 2009;25(16):2078-2079.
3. Cibulskis K, Lawrence MS, Carter SL, et al. Sensitive detection of somatic point mutations in impure and heterogeneous cancer samples. Nat Biotechnol. 2013;31(3):213-219.
4. Alexandrov LB, Stratton MR. Mutational signatures: the patterns of somatic mutations hidden in cancer genomes. Curr Opin Genet Dev. 2014;24:52-60.
5. Nik-Zainal S, Davies H, Staaf J, et al. Landscape of somatic mutations in 560 breast cancer whole-genome sequences. Nature. 2016;534(7605):47-54.
6. Rosenthal R, McGranahan N, Herrero J, Taylor BS, Swanton C. DeconstructSigs: delineating mutational processes in single tumors distinguishes DNA repair deficiencies and patterns of carcinoma evolution. Genome Biol. 2016;17:31.
7. Bellido F, Pineda M, Aiza G, et al. POLE and POLD1 mutations in 529 kindred with familial colorectal cancer and/or polyposis: review of reported cases and recommendations for genetic testing and surveillance. Genet Med. 2016;18(4):325-332.
8. Valle L, Hernández-Illán E, Bellido F, et al. New insights into POLE and POLD1 germline mutations in familial colorectal cancer and polyposis. Hum Mol Genet. 2014;23(13):3506-3512.
9. Castaño-Vinyals G, Aragonés N, Pérez-Gómez B, et al. Population-based multicase-control study in common tumors in Spain (MCC-Spain): rationale and study design. Gac Sanit. 2015;29(4):308-315.
10. Walsh MF, Ritter DI, Kesserwan C, et al. Integrating somatic variant data and biomarkers for germline variant classification in cancer predisposition genes. Hum Mutat. 2018;39(11):1542-1552.
11. Guha T, Malkin D. Inherited TP53 Mutations and the Li–Fraumeni Syndrome. Cold Spring Harb Perspect Med. 2017;7(4).
12. Shevelev IV, Hübscher U. The 3' 5' exonucleases. Nat Rev Mol Cell Biol. 2002;3(5):364-376.
13. Castellsagué E, Li R, Aligue R, et al. Novel POLE pathogenic germline variant in a family with multiple primary tumors results in distinct mutational signatures. Hum Mutat. 2018.
14. Hamzaoui N, Alarcon F, Leulliot N, et al. Genetic, structural, and functional characterization of POLE polymerase proofreading variants allows cancer risk prediction. Genet Med. 2020.
15. Rohlin A, Zagoras T, Nilsson S, et al. A mutation in POLE predisposing to a multi-tumour phenotype. Int J Oncol. 2014;45(1):77-81.
16. Vande Perre P, Siegfried A, Corsini C, et al. Germline mutation p.N363K in POLE is associated with an increased risk of colorectal cancer and giant cell glioblastoma. Fam Cancer. 2019;18(2):173-178.
17. Palles C, Cazier JB, Howarth KM, et al. Germline mutations affecting the proofreading domains of POLE and POLD1 predispose to colorectal adenomas and carcinomas. Nat Genet. 2013;45(2):136-144.

18. Spier I, Holzapfel S, Altmüller J, et al. Frequency and phenotypic spectrum of germline mutations in POLE and seven other polymerase genes in 266 patients with colorectal adenomas and carcinomas. Int J Cancer. 2015;137(2):320-331.
19. Wimmer K, Beilken A, Nustede R, et al. A novel germline POLE mutation causes an early onset cancer prone syndrome mimicking constitutional mismatch repair deficiency. Fam Cancer. 2017;16(1):67-71.
20. Feliubadaló L, Tonda R, Gausachs M, et al. Benchmarking of Whole Exome Sequencing and Ad Hoc Designed Panels for Genetic Testing of Hereditary Cancer. Sci Rep. 2017;7:37984.
21. Castellanos E, Gel B, Rosas I, et al. A comprehensive custom panel design for routine hereditary cancer testing: preserving control, improving diagnostics and revealing a complex variation landscape. Sci Rep. 2017;7:39348.
22. Aoude LG, Heitzer E, Johansson P, et al. POLE mutations in families predisposed to cutaneous melanoma. Fam Cancer. 2015;14(4):621-628.
23. Jansen AM, van Wezel T, van den Akker BE, et al. Combined mismatch repair and POLE/POLD1 defects explain unresolved suspected Lynch syndrome cancers. Eur J Hum Genet. 2015.
